# Nature Already Did the Screening: Drought-Driven Rhizosphere Recruitment Enables Inoculant Discovery in Tomato and Reveals a Candidate Novel *Paracoccus* Species

**DOI:** 10.3390/microorganisms14040747

**Published:** 2026-03-26

**Authors:** Kusum Niraula, Maria Leonor Costa, Lilas Wolff, Henrique Cabral, Millia McQuade, Lucas Amoroso Lopes de Carvalho, Daniel Silva, André Sousa, Juan Ignacio Vilchez

**Affiliations:** iPlantMicro Laboratory, Instituto de Tecnologia Química e Biológica (ITQB)-NOVA, Oeiras, 2780-157 Lisboa, Portugal; kniraula@itqb.unl.pt (K.N.); mlp.costa@campus.fct.unl.pt (M.L.C.); gzp650@alumni.ku.dk (L.W.); hamaralcabral@gmail.com (H.C.); milli.mcquade@itqb.unl.pt (M.M.); lucas.amoroso@unesp.br (L.A.L.d.C.); daniel.silva@itqb.unl.pt (D.S.); asousa@itqb.unl.pt (A.S.)

**Keywords:** drought-driven recruitment, rhizosphere microbiome, nature-guided screening, microbial inoculant, plant–microbe interactions

## Abstract

Drought is a major constraint on crop productivity, and microbial inoculants are increasingly explored to mitigate plant water stress. However, most inoculant discovery pipelines rely on trait-based screening performed outside the ecological context in which beneficial plant-microbe interactions naturally arise. In natural soils, drought-exposed plants can reshape the rhizosphere environment by altering carbon allocation and root exudation, thereby selectively recruiting microorganisms compatible with water-limited conditions and effectively performing an ecological pre-selection. Here, we captured this process during early seedling establishment and leveraged drought-driven rhizosphere recruitment as a nature-guided strategy to nominate bacterial inoculant candidates. Tomato seedlings were grown in natural agricultural soil microcosms under well-watered and drought-stressed regimes, and cultivable bacteria were comparatively isolated from rhizosphere and bulk soil fractions. Recruitment-prioritized isolates were subsequently characterized through biochemical assays and genome-informed analyses to provide functional and taxonomic context and were evaluated in early inoculation assays under water stress. Drought-recruited isolates displayed distinct plant-associated responses, and genome-scale taxonomy indicated that one drought-associated isolate represents a genomically distinct lineage within the genus *Paracoccus*. Together, these findings highlight drought-driven rhizosphere recruitment as an ecologically grounded framework for identifying stress-compatible bacterial candidates and uncovering previously undescribed rhizosphere diversity.

## 1. Introduction

Drought is among the most consequential climate-linked risks to agricultural productivity and food security, with increasing evidence that climate change is intensifying the frequency and severity of drought episodes in many cropping systems [1,2,3]. Because drought constrains plant growth through both direct hydric limitation and downstream metabolic disruption, there is strong interest in scalable strategies that enhance crop performance under water stress beyond genetic improvement or irrigation inputs alone [1,2,3,4].

Plant-associated microbiomes, particularly rhizosphere bacteria, can influence plant performance under stress through a range of microbial mechanisms, including modulation of plant stress signaling, production of extracellular polymers that influence soil microenvironments, and effects on root system architecture. These microbial traits can ultimately contribute to improved nutrient acquisition and plant resilience under adverse conditions [5,6,7,8]. In response to drought, plants not only adjust their internal physiology but also modify the chemical environment of the rhizosphere through changes in carbon allocation and the composition of root exudates. Root exudates comprise a complex mixture of organic acids, sugars, amino acids, secondary metabolites, and small signaling molecules released into soil [9,10,11,12]. Drought stress can alter both the quantity and quality of these exudates [11], shaping the availability of microbial substrates and chemical cues that influence rhizosphere community assembly [9,10,11,12,13,14]. Root exudates can recruit beneficial microbes through chemotaxis and by modulating microbial gene expression and activity, thereby affecting community composition and function in the rhizosphere. Although exudation-driven signaling is dynamic throughout plant development, early seedling establishment represents a particularly sensitive window, as young roots rapidly initiate microbial recruitment while rhizosphere communities are still assembling. Microbes that establish during this phase may exert disproportionate effects on subsequent rhizosphere trajectories and plant stress responsiveness [9,10,12].

This interplay between host exudation and microbial recruitment under stress has been conceptualized as an ecological communication process, including the plant “cry-for-help” hypothesis, whereby drought-induced exudate changes may contribute to the assembly of microbial partners associated with stress tolerance [15,16]. However, this interpretation is not exclusive, as drought-associated microbiome shifts may also reflect environmental filtering driven by changes in substrate availability, osmotic conditions, oxygen status, reduced diffusivity, or intrinsic microbial drought tolerance traits independent of plant signaling. Accordingly, drought-associated shifts in plant–microbiome assembly are likely shaped by both plant-mediated recruitment and direct ecological selection imposed by water limitation [17,18]. Despite growing mechanistic insight into drought-associated microbiome dynamics, translating these interactions into effective microbial inoculants remains challenging. Traditional inoculant discovery pipelines often deploy trait-first screening approaches in simplified laboratory settings that may not capture the ecological constraints required for persistence and function in natural soils [19,20,21]. In practice, this contributes to variable inoculant performance across environments, reflecting interactions among introduced strains, indigenous microbial communities, soil physicochemistry, and agronomic context [20,21,22].

While drought-driven recruitment can involve multiple microbial domains and kingdoms, including non-culturable bacteria, fungi, and archaea, we focus here on the cultivable bacterial fraction as a tractable and application-oriented entry point. We acknowledge that cultivation conditions capture only a subset of the rhizosphere microbiome and may distort relative abundance patterns, but they provide a practical framework for identifying candidate strains that can be readily propagated and experimentally validated. Using tomato seedlings grown in natural agricultural soil under controlled water limitation, we (i) isolated cultivable rhizosphere bacteria associated with drought exposure, (ii) prioritized candidates through targeted biochemical assays linked to plant interaction and drought-relevant traits, (iii) leveraged genome-based analyses to support functional potential and taxonomic placement of priority strains, and (iv) validated recruitment-informed candidates via early inoculation assays under water stress. We focused on an early establishment time window, when drought-induced shifts in exudation and recruitment may represent a critical inflection point for plant survival and resilience in natural soil. As an additional outcome of this recruitment-based screening, genome-scale taxonomy supported that one drought-enriched isolate represents a candidate novel species within the genus *Paracoccus*. Together, this work frames drought-driven recruitment not merely as an observational phenomenon but as a practical and ecologically informed screening pipeline for inoculant discovery.

## 2. Materials and Methods

### 2.1. Plant Material, Soil, and Experimental Design

Tomato (*Solanum lycopersicum* L.) seeds (Tres Cantos cultivar; Semillas Fitó, Barcelona, Spain) were used throughout the study. Seeds were surface-sterilized under aseptic conditions by immersion in 20% (*v*/*v*) commercial bleach solution for 7 min with gentle agitation, followed by 70% (*v*/*v*) ethanol for 3 min, and then rinsed thoroughly (≥3 times) with sterile double-distilled water (ddH_2_O) [23]. Sterilization efficacy was verified by plating aliquots of the final rinse water on LB agar and by imprinting sterilized seeds on LB agar plates; plates were inspected for microbial growth after incubation.

Sterilized seeds were pre-germinated on sterile, moistened paper towels in closed sterile containers at room temperature until radicle emergence. Five days post-germination, seedlings were transferred to pots containing natural agricultural soil collected from the Oeiras AGROTECH Campus experimental station (Portugal). The soil is classified as a fine-sandy soil with a pH of approximately 8.0, EC = 0.55 (measured with Soil Integrated Sensor 4001-BXSZD (Walfront; Shenzhen, China)), and organic matter content of 0.95% (carried out by the Soil Analysis Laboratory of the National Institute of Agricultural and Veterinary Research (INIAV) in Lisbon (Portugal)). Soil was homogenized and sieved to remove large debris while preserving its native physico-chemical and microbial characteristics, following common practice for controlled soil-based microbiome experiments [24,25,26]. Microcosms were maintained under greenhouse conditions (May 2025). A detailed soil characterization sheet is provided in the Figshare repository associated with this study. Drought stress was imposed during early establishment by applying two watering regimes: a well-watered control (maintaining approximately 70% of field capacity) and a drought treatment implemented by applying 30% of the irrigation volume used in the control treatment over a two-week period, followed by maintenance under reduced water availability. The total experimental duration was three weeks. Each treatment consisted of 10 biological replicates, with 3 seedlings per replicate.

### 2.2. Soil Microcosm Design for Recruitment-Based Culturomics

**Figure 1:**
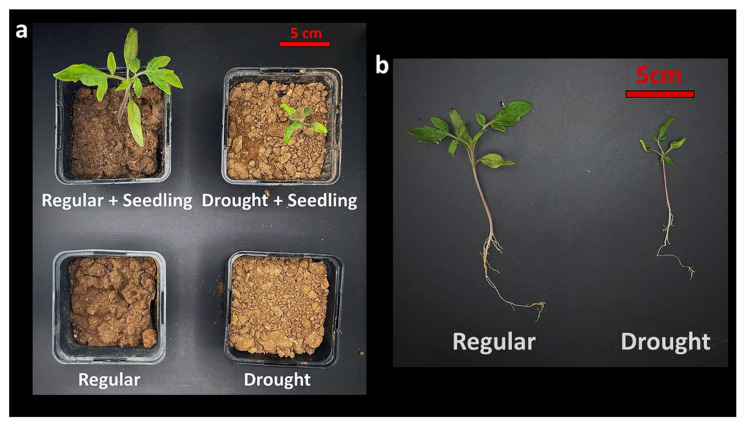
Soil microcosm framework to resolve rhizosphere recruitment during early seedling establishment under drought. (**a**) Overview of the soil microcosm conditions used to disentangle plant presence and drought effects within a constant soil background, including plant-free bulk soil controls (Regular and Drought) and tomato seedling-containing microcosms under well-watered (Regular + Seedling) and drought-stressed (Drought + Seedling) regimes. (**b**) Representative seedlings recovered at the end of the experimental period illustrating early developmental responses to contrasting water availability and providing the ecological context for defining cultivable rhizosphere bacterial populations during initial community assembly under stress.

To assess drought-driven microbial recruitment under controlled yet ecologically realistic conditions, soil microcosms were established using natural agricultural soil as the sole substrate. Microcosms consisted of standard pot systems filled with 0.40 L of homogenized soil and were assigned to one of three conditions: (i) soil without plants and well-watered conditions, (ii) soil without plants and drought conditions, (iii) soil with tomato seedlings under well-watered conditions, and (iv) soil with tomato seedlings subjected to drought stress (Figure 1). This design enabled direct comparison of cultivable bacterial populations across soil-only and plant-associated environments, while resolving drought-associated enrichment patterns within the same soil matrix. Microcosms were maintained under identical greenhouse conditions [27].

### 2.3. Rhizosphere and Bulk Soil Sampling

Bulk soil samples were collected at the beginning of the test (T = 0) and later, from plant-free microcosms to provide a baseline reference for soil-associated microbial communities and the physicochemical background of the native soil matrix. Bulk soil was collected from the central pot volume and homogenized prior to downstream processing. On the other hand, rhizosphere soil was collected in parallel at the end of the experimental period using a root-adhering soil collection approach. Seedlings were gently removed from the soil, and loosely attached soil was removed by shaking. Soil tightly adhering to the root surface was collected by brushing roots into sterile containers, operationally defining the rhizosphere fraction [24]. This paired sampling strategy supported inference of recruitment-driven enrichment by contrasting cultivable bacterial recovery in the rhizosphere against the surrounding soil background. This microcosm-based approach enables disentangling the effects of plant presence and drought stress on cultivable bacterial populations while preserving native soil complexity and physicochemical context [24,27].

### 2.4. Culturomics and Population-Level Discrimination of Bacterial Isolates

Cultivable bacterial communities were assessed using a culturomics-inspired approach, focusing on comparative recovery of bacterial populations across microcosm conditions rather than exhaustive enumeration. Soil suspensions were prepared from bulk soil and rhizosphere samples (root-adhering soil) using 0.25 g of material per sample, suspended in sterile 0.45% (*w*/*v*) NaCl solution, serially diluted, and plated exclusively on Luria–Bertani (LB) agar (per liter: 10 g NaCl, 5 g yeast extract, 10 g tryptone, and 15 g agar). Plates were incubated at 28 °C and monitored at 24 and 48 h. Colony-forming units (CFUs) were enumerated for each sampling point and expressed relative to the dry mass (mg) of the original soil material. This single-medium cultivation strategy was intentionally selected to prioritize robust, readily cultivable strains that can be propagated under standardized conditions, thereby facilitating comparative screening across treatments and supporting downstream scalability considerations relevant to potential bioinoculant development. Accordingly, this microcosm-based approach enables comprehensive coverage of the total rhizosphere microbiome, including fungal communities or nutritionally fastidious bacteria requiring specialized cultivation conditions. We acknowledge that LB-based recovery may bias community representation toward copiotrophic and fast-growing taxa; however, this selective recovery aligns with the application-oriented goal of prioritizing strains with high culturability and scalability potential.

Distinct colonies were selected based on morphology, pigmentation, and growth dynamics (consistent with standard microbiological criteria), and were purified by repeated streaking to obtain axenic cultures [28]. Purified isolates were preserved at −80 °C in 40% (*v*/*v*) glycerol. Isolates were catalogued according to their condition of recovery and classified as drought-enriched (isolated exclusively or predominantly from drought-stressed rhizosphere microcosms), plant-associated but drought-independent (recovered from both well-watered and drought-stressed rhizosphere microcosms), or soil-associated (recovered primarily from plant-free bulk soil microcosms). Population-level discrimination was performed by comparing isolate presence–absence patterns and relative recovery frequency across microcosm conditions, enabling identification of candidate taxa selectively associated with plant presence and drought stress. This comparative cultivation strategy prioritizes ecological association and recruitment signatures over absolute abundance and serves as an effective first-pass framework to nominate early inoculant candidates enriched under water limitation [29,30].

#### Morphological Grouping and Molecular Identification of Cultivable Isolates

Cultivable bacterial isolates recovered from soil microcosms were initially grouped based on colony morphology [28]. Representative isolates from each morphological group were selected for molecular identification to confirm taxonomic consistency and to reduce redundancy among isolates recovered across microcosm conditions, prioritizing morphotypes repeatedly observed across replicate plates and/or conditions.

Template DNA was obtained from individual colonies using a heat-shock extraction protocol, and DNA concentration/purity was evaluated by spectrophotometry (NanoDrop™ One, Thermo Scientific™, Agawam, MA, USA). Molecular identification was performed by PCR amplification of the bacterial 16S rRNA gene using either the V5–V8 hypervariable region (~700 bp) or the near full-length 16S rRNA gene (~1500 bp). The V5–V8 region was amplified using the universal primer set 799F (5′-AACMGGATTAGATACCCKG-3′) and 1392R (5′-GGTTACCTTGTTACGACTT-3′), whereas near full-length amplification employed primers 27F (5′-AGAGTTTGATCCTGGCTCAG-3′) and 1492R (5′-CTACGGCTACCTTGTTACGA-3′) [31,32]. PCR reactions were prepared using NZYTaq II 2× Master Mix (Nzytech, Lisbon, Portugal) and run in a Axygen^®^ MaxyGene II Thermal Cycler (Corning, New York, NY, USA) under the following cycling conditions: initial denaturation at 95 °C for 2 min; 40 cycles of denaturation at 95 °C for 3 s, annealing at 45 °C for 30 s, and elongation at 72 °C for 2 min; followed by a final extension step at 72 °C for 7 min. PCR amplification included no-template controls.

Amplification products were verified by electrophoresis on 1% agarose gels and visualized under UV illumination using a Benchmark myGel InstaView Complete Electrophoresis System with Blue LED Illuminator (VWR, Lisbon, Portugal). PCR products were purified and sequenced by Sanger sequencing (GENEWIZ, Azenta Life Sciences, Griesheim, Germany). Resulting sequences were quality-checked, trimmed, and assembled where necessary. Taxonomic assignment of isolates based on 16S rRNA gene sequences was performed using BLASTn (https://www.ncbi.nlm.nih.gov/geo/query/blast.html) against the NCBI database. Because 16S rRNA similarity alone may not always resolve closely related bacterial species, these identifications were considered preliminary and primarily used for genus-level classification and strain grouping. Definitive species-level identification would require phylogenetic or genome-based analyses. Closest matches were used to support genus-level identification and, where sequence resolution was sufficient, tentative species-level assignment. Isolates reported as “Unidentified” corresponded to cases in which amplification failed repeatedly or sequence quality was insufficient for reliable assignment. These identifications guided the selection of representative isolates for downstream genomic and functional analyses. This molecular identification step was used to validate morphology-based grouping and prioritize representative strains for follow-up characterization, rather than to infer community structure or quantitative abundance patterns across treatments.

The 16S rRNA gene sequences generated in this study have been deposited in GenBank under accession numbers PX985972–PX985973. In selected isolates, repeated Sanger sequencing attempts failed despite successful PCR amplification; therefore, whole-genome sequencing was performed only for strain Q, whose taxonomic identity could not be confidently resolved after repeated 16S rRNA gene amplification and Sanger sequencing. The remaining representative isolates were identified based on 16S rRNA gene sequence similarity against the NCBI database.

### 2.5. Genome Sequencing, Annotation, and Targeted Analysis

Genomic DNA from selected priority isolates was extracted using standard protocols suitable for whole-genome sequencing. Illumina paired-end sequencing was performed, generating high-quality short reads. Raw Illumina paired-end reads were subjected to quality control and preprocessing prior to assembly. Adapter sequences and low-quality bases were removed using Trimmomatic v0.39 with a sliding window quality cutoff of Q20 and a minimum read length of 50 bp. After filtering, a total of X paired reads were retained for downstream analysis. De novo genome assembly was performed using SPAdes v3.15.5 with default parameters optimized for bacterial genomes. Assembly quality was assessed using QUAST v5.2.0, which provided standard metrics including genome size, number of contigs, N50, and L50. To estimate sequencing depth and validate assembly consistency, filtered reads were mapped back to the assembled contigs using BWA-MEM v0.7.17, and coverage statistics were calculated using SAMtools v1.15 [33]. Sequencing reads were mapped back to the final assembly to assess overall coverage and consistency.

The draft genome assemblies and raw sequencing reads have been deposited in the NCBI under BioProject PRJNA1403472 (BioSample SAMN54674915; locus tag prefix ACYAOT). Whole-genome sequencing was undertaken for selected isolates following repeated failure of Sanger sequencing of 16S rRNA PCR products, despite consistent amplification, in order to ensure accurate taxonomic placement.

#### 2.5.1. Genome-Based Taxonomic Placement and Visualization

Genome-based phylogenomic analysis was performed using the Type Strain Genome Server (TYGS) platform, which implements genome-based distance phylogeny (GBDP) for taxonomic placement [34]. Whole-genome comparisons were performed against available type-strain genomes to identify the closest taxonomic relatives within the genus *Paracoccus* [24]. Intergenomic distances were calculated using the Genome BLAST Distance Phylogeny (GBDP) approach, and digital DNA-DNA hybridization (dDDH) values were estimated using recommended parameters. Phylogenomic relationships were inferred from genome-scale distance matrices and visualized as balanced minimum-evolution trees with branch support. Species-level relatedness was evaluated using the commonly accepted dDDH threshold of 70%. These analyses were used to assess taxonomic placement and species-level novelty without formal species description.

#### 2.5.2. Identification of Functional Gene Sets

Functional genes of interest were identified from the annotated genome and selected based on previously reported associations with plant-associated bacteria and stress tolerance mechanisms described in the literature. The analysis was intended as an exploratory survey of functional potential rather than a comprehensive functional characterization. Genes potentially associated with drought adaptation and rhizosphere competence were inferred from annotated genome features through literature-guided curation. Functional categories were defined based on database annotations, with emphasis on pathways previously implicated in rhizosphere colonization, stress tolerance, and plant–microbe interactions. Representative datasets from each functional category were selected for visualization and comparative purposes, including osmoprotection, oxidative stress response, motility and chemotaxis, surface polysaccharide production, and iron acquisition. This literature-guided approach was used to illustrate preliminary functional potential and support figure-based representations rather than to perform comprehensive genome-wide comparisons. A more detailed genomic characterization of this isolate is currently being conducted in the context of the formal description of a putative novel species, and therefore deeper functional analyses fall outside the scope of the present study.

### 2.6. Biochemical Screening for Plant-Associated and Drought-Relevant Traits

Based on their distribution across microcosm conditions, the rhizosphere isolates were selected for downstream characterization. Priority was given to identified isolates for biosafety reasons, evaluating their populational changes in enriched/appearing-only/neutral under drought conditions and defining the stress-independent strains used as internal controls. Selection aimed to balance ecological relevance with the feasibility of phenotypic and genomic analyses. Selected isolates were routinely cultured on LB agar or LB broth at 28 °C and maintained as glycerol stocks (20–30% *v*/*v*) at −80 °C.

The isolates were then screened for biochemical traits commonly associated with plant drought-stress mitigation using established qualitative or semi-quantitative assays [35,36]. Indole-related compound production was assessed using Salkowski reagent following standard protocols [37]. Siderophore production was evaluated using chrome azurol S (CAS) agar assays [38]. Biofilm formation was assessed using crystal violet staining in microtiter plates [39]. Moreover, proline production, one of the most relevant protectants against abiotic stresses, was evaluated following previously described approaches [40]. Where appropriate, assays were performed under both standard and drought-mimicking stress conditions to assess stress-dependent trait expression. All assays were conducted using at least 6 independent replicates, and results were interpreted comparatively among isolates to support candidate prioritization rather than as absolute quantitative measurements.

### 2.7. Quantification of Root Colonization

Root colonization was quantified following the protocol described by Vilchez et al. (2020) [23], with minor modifications to accommodate tomato seedlings and osmotic stress conditions. Water limitation was imposed by supplementing the medium with polyethylene glycol (PEG 6000) at 15% (*w*/*v*), a commonly used proxy to approximate drought-related reductions in water availability under controlled experimental conditions. Tomato seedlings were grown under sterile conditions as described above and used for colonization assays five days after germination. Individual strains were cultured in LB broth at 30 °C with shaking (220 rpm) for 24 h, harvested by centrifugation, washed, and resuspended in sterile 0.45% (*w*/*v*) NaCl solution to a standardized density of 10^6^ CFU mL^−1^. For osmotic stress treatments, PEG 6000 (15% *w*/*v*) was added to the bacterial suspension. Under sterile conditions, seedlings were transferred to tubes containing the inoculum and incubated overnight at 26 °C with slow shaking (80 rpm). After incubation, seedlings were rinsed three times with sterile ddH_2_O to remove loosely attached cells. Roots were excised, transferred to sterile tubes, and homogenized using sterile pestles. Homogenates were resuspended in 0.45% NaCl solution and serially diluted (10-fold series). Dilutions were plated on LB agar using the drop-plate method and incubated at 30 °C for 24 h. Colony counts were used to estimate colonization levels, expressed as CFU per root system or normalized to root dry weight (CFU g^−1^ root DW). Colonization was quantified for each strain under control and PEG-induced osmotic stress conditions using three biological replicates with five seedlings [36].

### 2.8. Early Inoculation Assays Under Water Stress

To validate the functional relevance of recruitment-informed screening, early inoculation assays were performed using selected priority isolates and applying protocols previously described [35,36]. Tomato seedlings were transferred to 0.5 L pots (filled with soil mix [turf:vermiculite, 1:1 *w*:*w*]), and each pot was inoculated with 40 mL of bacterial suspension adjusted to approximately 10^6^ CFU mL^−1^ [23]. Inoculation was performed via soil drench to ensure uniform application across treatments. Experimental treatments included non-inoculated controls, plants inoculated with drought-enriched isolates, and plants inoculated with strains not specifically enriched under drought (control). Following inoculation, seedlings were grown under greenhouse conditions, following the same drought regime described above. Ten biological replicates were prepared per condition.

Plant performance under water stress was evaluated by measuring plant height, root length, and dry biomass. Measurements were collected at 14 days after treatment, corresponding to peak stress exposure.

### 2.9. Statistical Analysis

All statistical analyses were conducted using GraphPad Prism v9.5.0. Data were tested for normality and homoscedasticity prior to analysis. Statistical analyses were performed using *Student’s t*-*tests* or one-way ANOVA depending on the comparison being evaluated. These tests were used to compare predefined treatment groups within each experimental condition (e.g., inoculated vs. non-inoculated seedlings within the same water regime). This approach was chosen to evaluate specific treatment contrasts rather than fitting a full factorial model. Biological replicates were treated as independent experimental units. Post hoc comparisons were performed using Tukey’s Honest Significant Difference (HSD). Significance thresholds were set at *p* < 0.05. Graphs were generated using GraphPad Prism v9.5.0 and R v4.5.2.

### 2.10. Data Availability

The datasets generated and analyzed during the current study are publicly available as follows. The 16S rRNA gene sequences obtained from representative cultivable isolates, corresponding to the V5–V8 hypervariable region or near full-length amplicons, have been deposited in the NCBI GenBank database under accession numbers PX985972–PX985973. The draft annotated genome sequence of *Paracoccus* sp. strain Q has been deposited in NCBI under BioProject PRJNA1403472 (BioSample SAMN54674915; locus tag prefix ACYAOT; Taxonomy ID: 267). The genome assembly is publicly accessible together with the corresponding raw sequencing reads in the NCBI Sequence Read Archive (SRA) under the same BioProject accession.

Quantitative datasets from biochemical and drought-related phenotypic assays, as well as the soil characterization sheet, are deposited in Figshare (DOI: 10.6084/m9.figshare.31431346), including raw replicate measurements, experimental metadata, and processed datasets used for statistical analysis. All additional data supporting the findings of this study are available from the corresponding author upon reasonable request.

## 3. Results

### 3.1. Population Dynamics of Cultivable Isolates Across Microcosm Conditions

**Figure 2:**
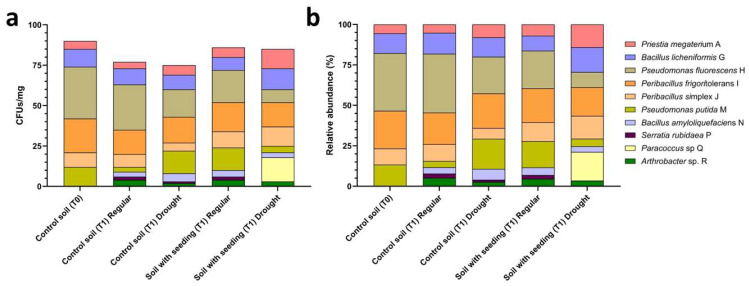
Recruitment dynamics of cultivable rhizosphere isolates across soil treatments. (**a**) Absolute abundance of cultivable bacterial isolates expressed as colony-forming units (CFU) per mg dry soil and (**b**) corresponding relative abundance (%) across soil treatments, including control soil at time 0 (T0), control soil at T1 under regular watering and drought, and soil with tomato seedlings at T1 under regular watering and drought. Stacked bars represent the contribution of individual isolates to the total cultivable community within each treatment. Isolate identities are indicated by color-coded segments as shown in the legend.

Cultivable bacterial populations differed across soil microcosm conditions in both total abundance and taxonomic distribution of cultured isolates (Figure 2). Absolute abundance decreased from 90 CFU g^−1^ dry soil at T0 to 77 CFU g^−1^ in plant-free soil at T1 under regular irrigation (−14%) and to 75 CFU g^−1^ under drought (−17%). In seedling-containing microcosms, total recoverable populations reached 86 CFU g^−1^ under regular irrigation (−4% vs. T0) but declined to 73 CFU g^−1^ under drought (−19%), indicating that water limitation reduced the cultivable fraction across both bulk and rhizosphere soils while rhizosphere populations under regular irrigation remained comparatively stable.

Relative recovery frequencies indicated differences in the distribution of cultured isolates across treatments. At T0, the cultivable fraction was dominated by *Pseudomonas fluorescens* H (35.6%) and *Peribacillus frigoritolerans* I (23.3%), together accounting for nearly 60% of recovered isolates, whereas other taxa were present at lower frequencies ranging from 3–13%. Temporal progression in plant-free soil resulted in moderate compositional changes. Under regular irrigation, *P. fluorescens* H decreased slightly to 30.4% (−15% relative change from T0), whereas drought exposure reduced its contribution further to 22.7% (−36% relative change from T0). In parallel, *Pseudomonas putida* M increased from 13.3% at T0 to 18.7% under drought (+40% relative increase), and Bacillota representatives collectively expanded their contribution compared with T0.

Rhizosphere samples showed a more even taxonomic distribution compared with bulk soil. Under regular irrigation, *P. fluorescens* H accounted for 23.3% of isolates (−34% relative to T0), followed by *P. putida* M (16.3%), *Peribacillus simplex* J (11.6%), and *Priestia megaterium* A (7.0%), reflecting reduced dominance of a single taxon relative to bulk soil. Under drought, this redistribution became more pronounced. *P. fluorescens* H declined to 11.0%, representing a threefold reduction relative to T0, while Bacillota representatives increased markedly, including *Peribacillus simplex* J and *Priestia megaterium* A, both reaching 16.4%. *Peribacillus frigoritolerans* I remained consistently represented across conditions, showing limited variation in relative abundance. A notable observation involved *Paracoccus* sp. Q, which was not recovered from bulk soil or well-watered rhizosphere samples and represented 20.5% of isolates recovered exclusively from drought-stressed rhizosphere microcosms.

These contrasting recovery patterns guided the selection of representative isolates for downstream biochemical characterization. *Priestia megaterium* A was prioritized due to its increased representation in seedling-containing microcosms, particularly under drought conditions. *Paracoccus* sp. Q was selected based on its exclusive recovery from drought-stressed rhizosphere samples, whereas *Peribacillus frigoritolerans* I was retained as a stress-independent comparator due to its consistent recovery across microcosm conditions.

### 3.2. Biochemical Profiling of Recruitment-Prioritized Isolates Highlights Complementary Plant-Associated and Drought-Relevant Traits

**Figure 3:**
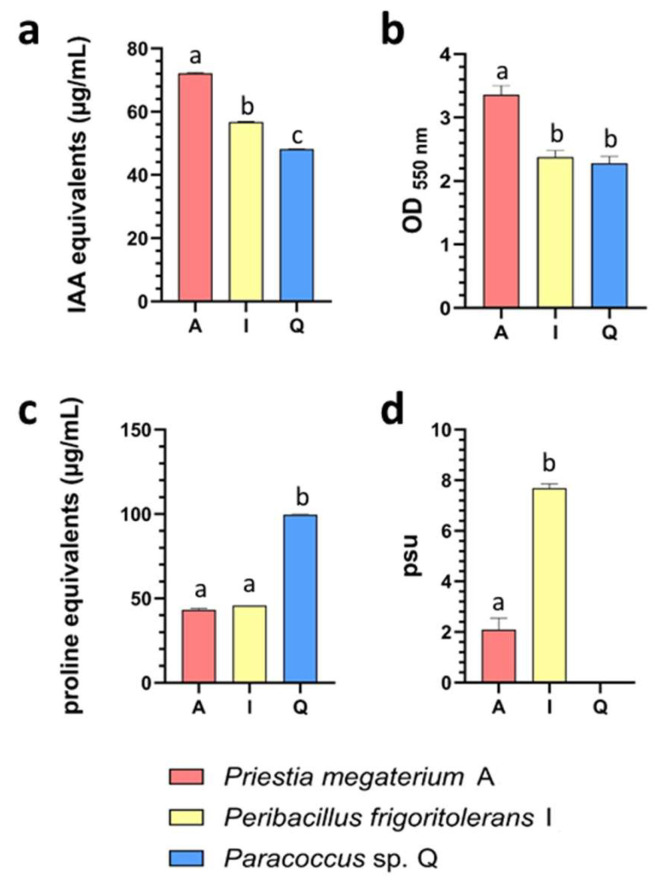
Biochemical characterization of recruitment-prioritized rhizosphere isolates. Biochemical traits associated with plant interaction and drought tolerance were assessed in representative isolates *Priestia megaterium* A, *Peribacillus frigoritolerans* I, and *Paracoccus* sp. Q. Auxin-related compound production is shown in panel (**a**) and expressed as IAA equivalents (µg mL^−1^). Biofilm formation capacity is presented in panel (**b**) following crystal violet staining and reported as optical density at 550 nm (OD_550_). Osmolyte-associated proline production is depicted in panel (**c**) and expressed as proline equivalents (µg mL^−1^). Siderophore production is shown in panel (**d**), quantified using the chrome azurol S (CAS) assay and expressed as percent siderophore units (psu). Bars represent mean values ± variation across biological replicates, and letters indicate statistically significant differences among isolates (*p* < 0.05).

To functionally characterize isolates displaying contrasting recovery patterns across microcosm conditions, representative strains *Priestia megaterium* A, *Peribacillus frigoritolerans* I, and *Paracoccus* sp. Q were screened for biochemical traits commonly associated with plant interaction and drought-relevant responses, including auxin-related compound production, biofilm formation, proline production, and siderophore production (Figure 3). These isolates were selected to represent increased rhizosphere abundance under drought (*P. megaterium* A), drought-specific recovery (*Paracoccus* sp. Q), and stress-independent persistence across treatments (*P. frigoritolerans* I).

Trait expression differed among isolates and across assays. Auxin-related compound production showed a graded distribution, with *P. megaterium* A displaying the highest IAA-equivalent levels. Relative to *P. megaterium* A, auxin production was reduced by approximately 20–25% in *P. frigoritolerans* I and by ~35% in *Paracoccus* sp. Q. A similar pattern was observed for biofilm formation, where crystal violet retention was strongest in *P. megaterium* A, while *P. frigoritolerans* I and *Paracoccus* sp. Q exhibited comparable but lower levels (~25–30% lower).

In contrast, proline production displayed an inverse distribution. Here, *Paracoccus* sp. Q showed the highest levels of proline equivalents, exceeding those detected in *P. megaterium* A and *P. frigoritolerans* I by more than twofold. Siderophore production further distinguished the isolates, with *P. frigoritolerans* I exhibiting the strongest CAS reactivity; siderophore units (psu) were roughly threefold higher than those observed in *P. megaterium* A, whereas activity was not detected in *Paracoccus* sp. Q under the tested conditions. Together, these results indicate a differential distribution of biochemical traits across the selected isolates, revealing distinct phenotypic profiles within the candidate set.

### 3.3. Root Colonization of Tomato Seedlings

**Figure 4:**
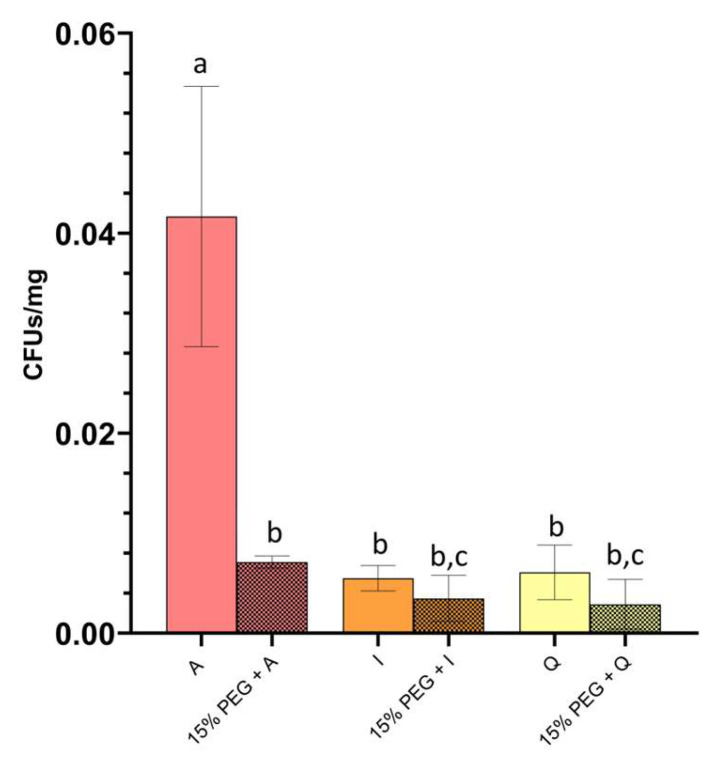
Root colonization of tomato seedlings by selected isolates. Root colonization by isolates represented by red bars (*P. megaterium* A), orange bars (*P. frigoritolerans* I), and yellow bars (*Paracoccus* sp. Q), quantified as colony-forming units (CFU) per mg root dry weight. Solid bars indicate colonization under regular conditions, whereas patterned bars represent colonization in the presence of 15% PEG as a drought-mimicking, osmotic treatment. Bars correspond to mean values and error bars indicate standard deviation of biological replicates (n = 5). Different letters denote statistically significant differences among treatments according to one-way ANOVA followed by a post hoc test (*p* < 0.05).

Root colonization differed among isolates and was affected by PEG-induced osmotic stress (Figure 4). Under control conditions, plants exposed to *P. megaterium* A displayed the highest colonization levels, exceeding those observed for *P. frigoritolerans* I and *Paracoccus* sp. Q by approximately 7.6-fold and 6.8-fold, respectively, whereas *P. frigoritolerans* I and *Paracoccus* sp. Q showed comparable colonization.

PEG exposure reduced colonization across all isolates, although the magnitude of reduction differed among treatments. Colonization by *P. megaterium* A decreased markedly under PEG, reaching approximately 0.17-fold of control levels (~83% reduction). In contrast, colonization by *P. frigoritolerans* I declined more moderately to 0.63-fold of control levels, while colonization by *Paracoccus* sp. Q decreased to 0.47-fold of control levels.

As a result, while *P. megaterium* A displayed substantially higher colonization under control conditions, it also exhibited the strongest proportional decline under PEG stress. Strains *P. frigoritolerans* I and *Paracoccus* sp. Q maintained a larger fraction of their control colonization levels, resulting in reduced differences among isolates under osmotic stress. These patterns describe differential colonization dynamics across isolates under osmotic conditions and provide contextual information for subsequent plant growth assays.

### 3.4. Early Inoculation Assays Support Recruitment-Informed Candidate Prioritization Under Drought

**Figure 5:**
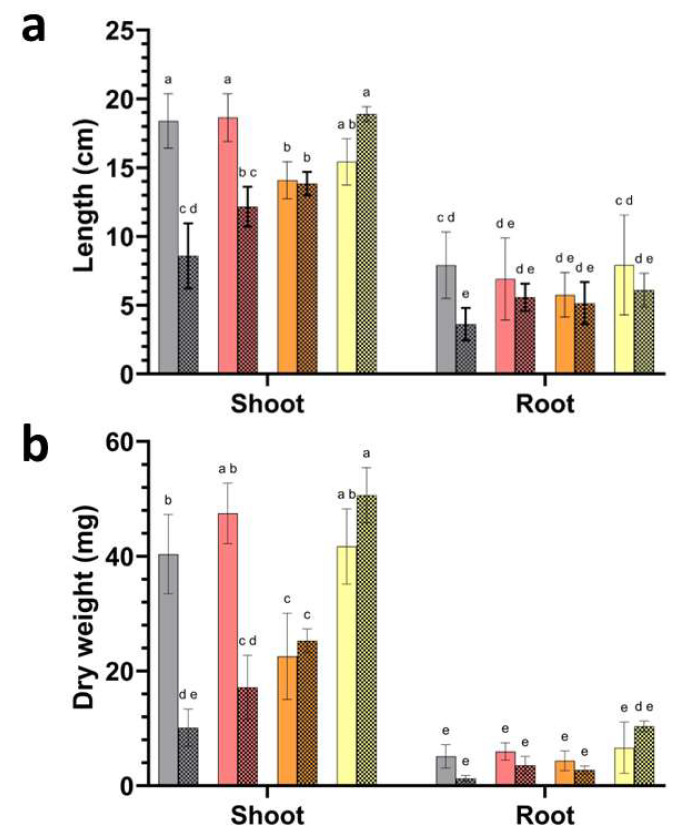
Plant growth responses to bacterial inoculation under regular watering and drought. (**a**) Shoot and root length and (**b**) shoot and root dry weight of tomato seedlings grown under regular watering or drought conditions following inoculation with isolates represented by grey bars (non-inoculated control), red bars (*P. megaterium* A), orange bars (*P. frigoritolerans* I), and yellow bars (*Paracoccus* sp. Q). Solid bars correspond to plants grown under regular watering, whereas patterned bars indicate drought-exposed treatments. Bars represent mean values and error bars indicate standard deviation of biological replicates (n = 5). Different letters denote statistically significant differences among treatments within each panel and organ (one-way ANOVA with post hoc test, *p* < 0.05).

Plant size and biomass differed across watering regimes and inoculation treatments. Plant biomass and length measurements revealed strong treatment-dependent differences under drought stress (Figure 5a,b). In non-inoculated plants, drought reduced shoot dry weight from 45.4 mg under regular watering to 17.6 mg, corresponding to an approximate 61% decrease. Root dry weight showed a similar decline, decreasing from 5.4 mg to 2.0 mg.

Inoculation altered these responses across treatments. Under drought, plants inoculated with *P. megaterium* A reached 17.1 mg shoot biomass, remaining within the range observed for drought-stressed controls and lower than plants grown under regular watering with the same inoculation (53.5 mg). Plants inoculated with *P. frigoritolerans* I displayed intermediate shoot biomass under drought (25.4 mg under regular watering), while the highest shoot biomass under drought was recorded in plants inoculated with *Paracoccus* sp. Q (50.4 mg), exceeding values observed in drought-stressed controls and approaching those observed under regular watering within the same inoculation group (40.2 mg). Root biomass followed comparable patterns. Under drought, plants inoculated with *P. megaterium* A reached 3.6 mg root biomass, whereas drought-stressed controls remained at 2.0 mg. The highest root biomass under drought was observed in plants inoculated with *Paracoccus* sp. Q (9.1 mg), exceeding values observed in non-inoculated drought treatments and regular watering controls (5.4 mg).

Shoot length measurements mirrored biomass responses. In non-inoculated plants, drought reduced shoot length from 18.4 cm to 8.6 cm. Under drought, plants inoculated with *P. megaterium* A reached 12.2 cm shoot length, whereas plants inoculated with *P. frigoritolerans* I reached 13.8 cm. The greatest shoot length under drought was observed in plants inoculated with *Paracoccus* sp. Q (18.8 cm), approaching values measured under regular watering within the same inoculation group (15.4 cm). Root length responses were more variable and showed smaller treatment effects. Drought reduced root length from 7.9 cm to 3.6 cm in non-inoculated plants. Under drought, plants inoculated with *P. megaterium* A reached 5.6 cm root length, while plants inoculated with strain I reached 5.1 cm. Plants inoculated with *Paracoccus* sp. Q reached 7.1 cm root length under drought, remaining within the range observed under regular watering (7.9 cm).

**Figure 6:**
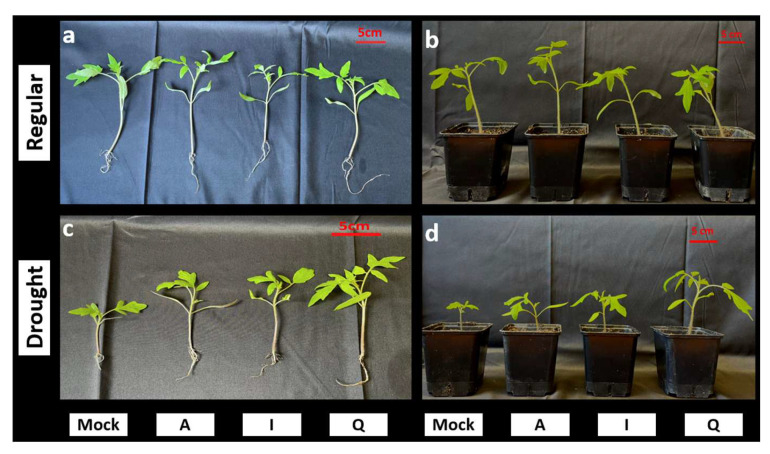
Representative phenotypes of tomato seedlings treated with the selected candidate strains. Representative images of tomato seedlings grown under regular watering (**a**,**b**) and drought conditions (**c**,**d**) following inoculation with the indicated treatments (Mock, non-inoculated control; A, *P. megaterium* A; I, *P. frigoritolerans* I; Q, *Paracoccus* sp. Q). Panels (**a**,**c**) show uprooted seedlings to visualize shoot and root architecture, whereas panels (**b**,**d**) show seedlings grown in pots. Images correspond to representative individuals from independent biological replicates included in the quantitative analyses. Scale bars represent 5 cm.

Representative seedlings illustrated these quantitative trends, with drought-stressed plants inoculated with *Paracoccus* sp. Q displaying visibly greater shoot development compared with other drought treatments (Figure 6). Together, these measurements demonstrate distinct quantitative growth responses among inoculation treatments under drought.

### 3.5. Targeted Genomic Analysis Supports Functional Potential and Taxonomic Novelty of a Drought-Recruited Paracoccus Isolate

Given its drought-associated recovery pattern and performance in early inoculation assays, strain Q was selected for genome-informed characterization. The draft genome comprised 4.0 Mb distributed across 78 contigs, with a GC content of 66.55% and 3798 predicted protein-coding sequences, together with 47 tRNA genes and three rRNA genes. These features support the classification of strain Q as a high-quality draft genome suitable for functional interpretation.

**Figure 7:**
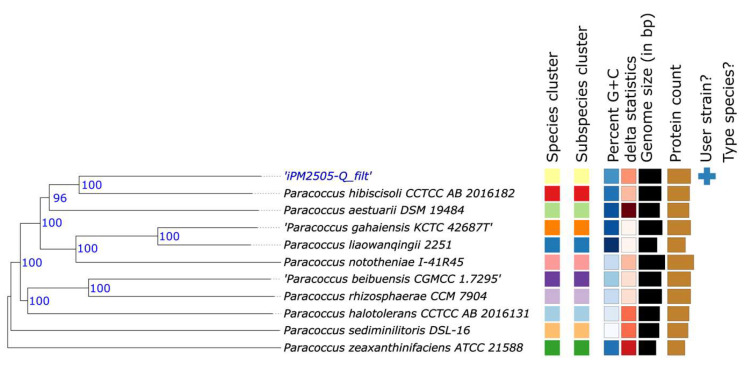
Genome-based phylogenetic placement of drought-recruited *Paracoccus* sp. Q. Phylogenomic tree inferred from TYGS analysis showing the position of strain Q relative to representative *Paracoccus* species. Bootstrap values are indicated at nodes. Colored blocks represent genome-associated metadata. The tree supports assignment of strain Q to the genus *Paracoccus*.

Genome-based taxonomic placement assigned strain Q to the genus *Paracoccus* (Figure 7). Digital DNA–DNA hybridization (dDDH) values relative to available *Paracoccus* type strains were well below the accepted species delineation threshold, with the highest value observed relative to *Paracoccus hibiscisoli* (31.1%), suggesting that strain Q represents a genomically distinct lineage within the genus and may correspond to a candidate novel species-level taxon.

**Figure 8:**
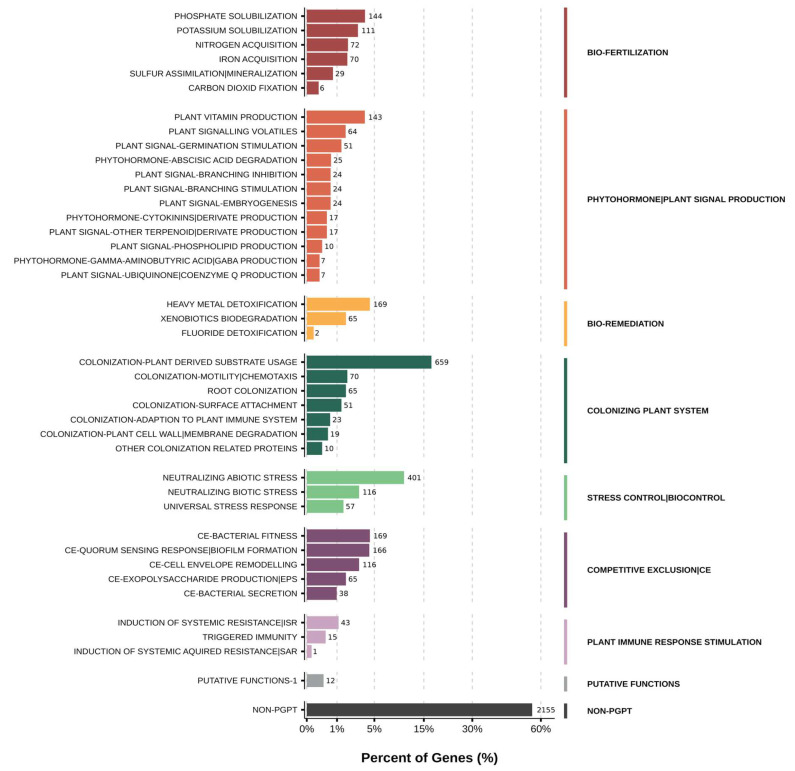
Functional repertoire of the drought-recruited *Paracoccus* sp. Q genome. Distribution of predicted gene functions associated with plant interaction, colonization, stress response, nutrient mobilization, and competitive traits based on PLaBAse annotation. Bars represent the proportion of genes assigned to each functional category within the draft genome.

Functional annotation revealed gene repertoires consistent with persistence in water-limited rhizosphere environments (Figure 8). Multiple osmoprotection pathways were detected, including trehalose biosynthesis via both the *otsA*/*otsB* and *treY*/*treZ* routes, as well as an ectoine biosynthesis cluster (*ectABC*) and compatible-solute transport systems. Genes linked to glycine betaine and choline uptake further expanded the repertoire of osmolyte-associated functions.

A comprehensive oxidative stress response network was also identified, including catalases (*katE*, *katG*), superoxide dismutases (*sodA*, *sodB*), and peroxiredoxin-associated functions, supporting resilience under drought-associated oxidative stress. Genes related to motility and chemotaxis were prominent, including structural and regulatory components of the flagellar apparatus and canonical chemotaxis signaling proteins (*cheA*, *cheY*, *cheW*, *cheR*, *cheB*), together with multiple methyl-accepting chemotaxis proteins.

Surface-associated persistence functions were represented by polysaccharide-related pathways, including cellulose synthesis (*acsAB*) and alginate-associated functions, together with additional biofilm-associated genes. The genome also encoded nutrient mobilization and acquisition traits, including a PQQ-dependent glucose dehydrogenase module (*pqqABCDE* and *gcd*) linked to mineral phosphate solubilization, phosphate uptake and regulation systems, and transporters for essential ions and micronutrients.

Canonical PGPR markers associated with ethylene modulation and hormone biosynthesis were not supported at the genomic level, as ACC deaminase genes were not detected in the draft annotation. These observations suggest that the drought-associated performance of strain *Paracoccus* sp. Q may be linked primarily to stress tolerance, rhizosphere persistence, and nutrient-related functions rather than classical hormone modulation pathways. These annotations indicate potential functional traits inferred from gene presence rather than demonstrated activity under drought conditions.

## 4. Discussion

The rhizosphere represents a critical interface through which plants interact with their microbial environment, and drought can influence ecological processes occurring within this interface. Previous studies have suggested that drought-associated shifts in rhizosphere microbial communities may involve plant-mediated changes that alter the chemical and physical soil environment, thereby influencing microbial recruitment and persistence [13,15,41]. Such processes have been proposed to contribute to restructuring rhizosphere communities under water limitation, although these mechanisms were not directly measured in the present study and are discussed here as potential explanations consistent with prior observations [13,41,42]. Alterations in root exudation patterns under water limitation modify substrate availability and signaling landscapes, thereby restructuring microbial assembly and activity in the rhizosphere [11,40]. Although such recruitment dynamics occur throughout plant development, our results demonstrate that recruitment signatures can be captured and operationalized already at the seedling stage, when microbiome assembly remains highly responsive to environmental cues and host-derived signals [43,44].

A central insight emerging from this work is that drought itself can function as an ecological filter. In natural soil, drought narrowed the cultivable candidate space while enriching a consistent subset of cultured bacteria recovered from stressed seedlings under the conditions tested [13,45,46]. This narrowing effect is conceptually important because it positions the plant–soil system as an initial selection layer that integrates stress tolerance, rhizosphere competence, and host compatibility before any laboratory characterization. Similar stress-associated patterns have been reported in community-level drought microbiome studies, where taxa characterized by osmotic resilience, metabolic flexibility, and surface-associated lifestyles tend to be more frequently detected under water-limited conditions [47,48]. However, such ecological filtering has rarely been translated into isolate-level discovery strategies, and our study demonstrates how recruitment signatures can be operationalized to guide candidate prioritization. Although the study relied on a single agricultural soil, this design intentionally minimized edaphic variability to enable the resolution of recruitment signatures attributable to plant presence and water limitation. Future multi-site validation will be necessary to assess the generality of recruitment-informed candidates across soil types and climatic contexts. The use of a single cultivation medium further constrained isolate recovery; however, this selective window was consistent with the application-oriented focus of identifying robust, readily cultivable strains. Expanding cultivation diversity in future work may reveal additional recruitment-associated taxa with complementary functional traits.

The distribution of stress-associated phenotypes among drought-recruited isolates further supports the value of recruitment-informed discovery, although these patterns were not formally assessed for statistical coherence across isolates. Phenotypes associated with rhizosphere persistence and stress adaptation, including biofilm formation, osmolyte-related responses, and iron acquisition strategies, were observed across the selected isolates. Importantly, these traits were not used as screening criteria but emerged as descriptive features of isolates already selected through recruitment. This distinction is particularly relevant given growing recognition that classical PGPR trait screening often fails to predict inoculant establishment or performance in complex soils. [49,50]. By integrating ecological compatibility upstream of functional characterization, recruitment-based pipelines may reduce the disconnect between laboratory trait expression and soil-based functionality that has historically limited microbial inoculant translation.

Root colonization assays provided additional insight into strain-specific interaction dynamics under osmotic stress. Although *Priestia megaterium* A exhibited higher colonization under non-stress conditions, BLASTn searches returned the highest similarity scores and the majority of top taxonomic matches to *P. megaterium*; however, closely related species such as *Peribacillus aryabhattai* may display nearly identical 16S rRNA sequences, which can limit species-level resolution using this marker alone [51]. Its pronounced proportional decline under PEG contrasted with the comparatively stable colonization of strains *P. frigoritolerans* I and *Paracoccus* sp. Q [52,53,54,55,56,57]. Interestingly, *Peribacillus frigoritolerans* has previously been reported as a plant-associated bacterium with potential roles in plant growth promotion and drought tolerance, including improved plant performance under water-limited conditions [55,56]. These observations suggest that colonization magnitude and tolerance to PEG-induced osmotic stress may represent distinct dimensions relevant to bacterial persistence under reduced water availability. Previous work has shown that beneficial effects on plant performance do not necessarily correlate with maximal root colonization density but may instead depend on metabolic compatibility, spatial niche occupation, or functional interactions within rhizosphere communities. However, because PEG-based assays impose osmotic stress in an axenic liquid system, these results should be interpreted as indicators of stress tolerance potential rather than direct evidence of rhizosphere resilience under soil drought conditions [5,58]. Accordingly, colonization data in this study provide contextual characterization of persistence dynamics rather than predictive screening metrics.

Early inoculation assays offered functional validation of the recruitment-informed framework. Drought reduced plant growth and biomass in non-inoculated seedlings, consistent with established impacts of water limitation on early developmental processes and carbon allocation. Inoculated treatments displayed distinct quantitative responses, with *Paracoccus* sp. Q consistently associated with the strongest plant performance under drought conditions. Although causality cannot be inferred from these assays alone, the observed responses indicate that recruitment-informed candidate selection can translate into measurable phenotypic differences during early establishment. This developmental window is increasingly recognized as a critical phase for microbiome-mediated modulation of plant stress resilience, as early colonizers may shape subsequent community assembly trajectories and functional interactions [43,59,60]. The inoculation assays were conducted during early seedling establishment, a deliberately short experimental window selected to capture recruitment-informed effects during a highly plastic developmental phase. While longer-term and field-based assessments will be required to evaluate persistence and agronomic relevance, early-stage responses provide a sensitive proxy for candidate prioritization.

Genome-informed characterization provided further ecological context for the drought-recruited *Paracoccus* isolate. For strain Q, selected stress-associated phenotypes were empirically confirmed through biochemical assays, whereas additional traits related to osmoprotection, oxidative stress response, chemotaxis, and nutrient acquisition were inferred from genome annotation and should be interpreted as potential functional capacities rather than experimentally validated activities. Background information discussed for the genus *Paracoccus* is provided for ecological context and does not constitute strain-specific evidence. Functional annotations revealed gene repertoires associated with osmoprotection, oxidative stress mitigation, chemotaxis, and nutrient mobilization, pathways widely implicated in microbial adaptation to drying soils and rhizosphere persistence [9,35,40,48,61]. Notably, canonical hormone-modulation genes were not detected in the current draft genome annotation. However, because the genome assembly represents a draft, the apparent absence of specific genes should be interpreted cautiously and may reflect assembly fragmentation, annotation gaps, or gene divergence. This observation nevertheless suggests that beneficial plant responses associated with this strain may involve alternative ecological mechanisms such as stress buffering, metabolic cross-feeding, or resource mobilization. Such interpretations align with emerging perspectives emphasizing that plant growth promotion in natural soils often reflects complex ecological interactions rather than single-trait mechanisms [8,44,62,63].

The recovery of a *Paracoccus* strain representing a candidate novel species among drought-associated isolates illustrates a potential outcome of recruitment-informed approaches: the identification of ecologically relevant microbial diversity within the cultivable fraction. Members of the genus *Paracoccus* are widely distributed across soil and plant-associated habitats and are known for considerable metabolic versatility, including traits related to stress tolerance, denitrification capacity, and diverse carbon utilization pathways. Although this observation derives from a single experimental system, it highlights how recruitment-informed screening may help uncover functionally relevant taxa associated with drought-affected rhizosphere conditions [51,53]. However, their roles in drought-associated rhizosphere interactions remain comparatively underexplored. The detection of a potentially novel lineage exclusively within drought-stressed rhizosphere samples suggests that stress-driven recruitment can expose functionally important microbial taxa that may be underrepresented in culture collections and trait-based screening pipelines. Culturomics studies have similarly demonstrated that selective environmental contexts can facilitate the recovery of previously undescribed taxa with specialized ecological functions [29,30,64,65].

Collectively, these findings support a conceptual shift in microbial inoculant discovery toward ecology-informed frameworks. Recruitment-based approaches position candidate strains as outcomes of plant-driven selection processes rather than externally optimized inputs, aligning with growing calls to incorporate ecological realism into microbiome applications in agriculture [66,67,68]. By observing how plants restructure their microbial environment under stress and redeploying those interactions experimentally, it becomes possible to identify microbial partners refined through repeated plant–soil interactions. In this sense, recruitment-guided discovery can be interpreted as a strategy that leverages host-mediated ecological filtering operating at the rhizosphere interface to inform inoculant design. The identification and functional characterization of *Paracoccus* sp. Q exemplify this perspective. Its drought-associated recruitment pattern, plant performance outcomes, and genomic features collectively suggest compatibility with water-limited rhizosphere conditions, while its potential taxonomic novelty underscores the exploratory value of recruitment-based pipelines. More broadly, this work illustrates how early-stage plant-microbe interactions can be captured and translated into candidate discovery strategies that connect ecological observation with applied microbial screening. Because the experiments were conducted during a short developmental window at the seedling stage, the plant responses observed here likely reflect early interaction dynamics that may include transient effects such as osmotic buffering or nutrient priming rather than stable long-term colonization. Future studies integrating exudate profiling, community-resolved sequencing, and multi-season validation will be required to clarify recruitment mechanisms and evaluate whether recruitment-informed candidates maintain their performance across longer developmental stages and diverse environmental contexts.

## 5. Conclusions

This study demonstrates that drought-driven rhizosphere recruitment can be leveraged as an ecologically informed framework to identify readily cultivable bacterial candidates with functional relevance under water stress. By integrating natural soil microcosms, comparative cultivation, biochemical and colonization characterization, genome-informed analysis, and early seedling-stage validation, our results are consistent with drought acting as an ecological filter that narrows the candidate space and enriches bacteria compatible with drought-altered rhizosphere conditions. Although exploratory, this recruitment-centered approach provides an alternative to conventional trait-first inoculant discovery pipelines by embedding ecological compatibility upstream of functional evaluation. The recovery of a drought-associated *Paracoccus* isolate representing a genomically distinct lineage further illustrates the capacity of recruitment-guided strategies to reveal previously undescribed, stress-associated microbial diversity while identifying candidates with measurable plant-associated effects.

Together, these findings support a perspective in which microbial inoculant discovery is grounded in plant-driven ecological selection and rhizosphere feedback processes. By aligning candidate identification with naturally occurring plant–microbe interactions, recruitment-informed pipelines may contribute to the development of microbiome-based solutions that are ecologically compatible and functionally relevant under water limitation.

## Data Availability

Genome sequence data generated in this study have been deposited in NCBI under BioProject accession number PRJNA1403472 (BioSample SAMN54674915; locus tag prefix ACYAOT). The draft genome assembly of *Paracoccus* sp. strain Q, including structural and functional annotation, is publicly available in GenBank under the same BioProject, together with the corresponding raw sequencing reads in the NCBI Sequence Read Archive (SRA). Partial 16S rRNA gene sequences of representative isolates (V5–V8 region or near full-length amplicons) have been deposited in GenBank under accession numbers PX985972–PX985973. Quantitative datasets from biochemical and drought-related phenotypic assays, including raw replicate measurements, experimental metadata, and processed datasets used for statistical analyses, are publicly available in Figshare (DOI: 10.6084/m9.figshare.31431346). All additional data supporting the findings of this study are available from the corresponding author upon reasonable request.
